# Composition of the Fecal Microbiota of Piglets at Various Growth Stages

**DOI:** 10.3389/fvets.2021.661671

**Published:** 2021-07-15

**Authors:** Yang Yang, Yadan Liu, Juan Liu, Haizhen Wang, Yulong Guo, Min Du, Chunbo Cai, Yan Zhao, Chang Lu, Xiaohong Guo, Guoqing Cao, Zhibian Duan, Bugao Li, Pengfei Gao

**Affiliations:** ^1^College of Animal Sciences, Shanxi Agricultural University, Taigu County, China; ^2^Department of Animal Sciences, Washington State University, Pullman, WA, United States; ^3^College of Veterinary Medicine, Shanxi Agricultural University, Taigu County, China

**Keywords:** piglet, fecal microbiota, growth and development, 16S rRNA sequencing, mashen pig, jinfen white pig

## Abstract

Gastrointestinal (GI) microbiota play an important role in promoting growth in piglets. However, studies on microbiota composition at various growth stages are lacking. We measured body weights of Jinfen White and Mashen piglets every 7 days and collected their fecal samples by rectal swabbing at nine time points during suckling (1–28 days) and nursery (35–70 days) stages to gain insight into microbiota variability during piglet growth. The fecal microbiota were characterized via 16S rRNA gene sequencing to analyze the effects of microbial diversity on piglet growth and development preliminarily. The results showed that although the two breeds of piglets have similar body weights at birth, weaned Jinfen White piglets demonstrated a significantly greater body weight and daily weight gain than weaned Mashen piglets (*P* < 0.01). A total of 1,976 operational taxonomic units (OTUs) belonging to 27 phyla and 489 genera were uncovered, in which the highest numbers of OTUs belong to the phyla Firmicutes and Bacteroidetes. *Lactobacillus, Bacteroides*, and *Prevotellaceae* NK3B31 groups accounting for 12.4, 8.8, and 5.8% of OTUs, respectively, showed relatively high abundance at the genus level. Nine sampling time points were divided into three growth stages, namely, immediate postfarrowing (1 day old), suckling (7, 14, and 21 days old), and nursery (28, 35, 49, 63, and 70 days old), on the basis of the results of microbial diversity, principal coordinate, and co-occurrence network analyses. In addition, it identified 54 discriminative features in the microbiota between two breeds of piglets by LEfSe analysis, in which 17 genera enriched the microbiota community of Jinfen White piglets. Finally, abundances of 29 genera showed significant positive correlations with body weights and daily weight gain of piglets. Conversely, abundances of 12 genera demonstrated significant negative correlations with body weights of piglets. The results of our study will provide a theoretical basis for succession patterns in fecal microbiota of piglets and suggest the need for meticulous management of piglets in pig production.

## Introduction

The gastrointestinal (GI) tract is the main reservoir for microbial communities containing abundant (10^13^-10^14^) microorganisms (1). The GI microbiota is predominantly composed of anaerobic bacteria but also contains fungi, archaea, and viruses (2). Microorganisms living in GI tracts of animals contribute to a complex microecosystem that can exert a profound impact on the animal's GI health and development (3). This mutualistic relationship has developed between microorganisms and animals over hundreds of millions of years. Health or onset of disease in animals is associated with microbial community composition and GI tract structure. Increasing evidence indicates that obesity and diabetes, which are risk factors for cardiovascular disease, are influenced by GI microbiota; thus, the microbiota may serve as a novel drug target for treatment (4, 5).

Digestive enzymes in pigs process starch, fats, and proteins, and their endocrine systems are similar to those of humans. Therefore, pigs are good animal models for exploring characteristics of the GI microbiota in humans. Firmicutes and Bacteroidetes found in GI tracts of pigs contribute to the overall health of the animal through various means (6). Firmicutes is highly abundant in the pig GI tract and involved in maintaining energy balance in the body (7, 8). Bacteroides play important roles in promoting health by producing butyrate, which activates T cell-mediated immune responses that subsequently limit GI tract colonization by potentially pathogenic bacteria (9). Proteobacteria also play important roles in promoting GI health, and its increased abundance can trigger inflammatory responses through the disruption of the GI microbiota equilibrium (10). *Prevotella* facilitates the degradation of proteins and carbohydrates and not only participates in polysaccharide degradation and amino acid metabolism in the host but also affects the production of intramuscular fats and storage of hepatic glycogen in pigs (10, 11).

Mashen pigs, an indigenous breed from Shanxi Province of China, are characterized by high fertility, excellent meat quality, and high stress resistance but slow growth rate. Jinfen White pigs are crossbred from various parental breeds, including Mashen (6.25%), Taihu (3.125%), Danish Landrace (40.625%), and Large White (50%) pigs. These pigs inherited excellent meat quality and high stress resistance from Mashen pigs while demonstrating the added benefit of a higher growth rate. Breed differences among pigs may affect the GI microbiota composition (12, 13), which in turn can influence the animal's productivity and ability to resist stress (14). We analyzed GI microbiota dynamics in Mashen and Jinfen White piglets in this study by observing differences in microbial diversity in their fecal microbiota. We also examined the effects of GI microorganisms on piglet growth rates and investigated patterns of colonization, development, and maturation of GI microorganisms in these piglets. Furthermore, we evaluated differences in specific bacterial genera between the two breeds. This study can provide a theoretical basis for further investigation of GI microbiota dynamics in pigs and insight into the effect of the microbiota on pig productivity.

## Materials and Methods

### Experimental Animal Feeding and Sample Collection

All animal procedures were performed in strict accordance with the Code of Ethics of the World Medical Association (http://ec.europa.eu/environment/chemicals/lab_animals/legislation_en.htm). The management and design of the experiment were kept to animal care rules approved by the Animal Ethics Committee of Shanxi Agricultural University (Shanxi, China). The Ethics Committee agreement number is SXAU-EAW-P002003. Experimental animals were maintained at the Datong Pig Breeding Farm (Shanxi Province, China) in accordance with Feeding Standards of Swine (NY/T 65-2004) of the Agriculture Industry Standards of China. A total of six healthy Mashen and Jinfen White sows (three of each breed) of the same age were selected for synchronized estrus induction and mating. Each sow was individually penned, and probiotics, antibiotics, or other drugs were excluded from their diets. Their piglets were offered creep feed on day 14 and weaned on day 28 after farrowing, at which point the animals were relocated to a nursery pen. The piglets entered the fattening or growth–finishing stage after the nursery stage by day 70 postfarrowing.

Subjects were weighed at 9:00 a.m. on days 1, 7, 14, 21, 28, 35, 49, 63, and 70 after farrowing, and fresh fecal samples from 24 (12 of each breed) piglets with a similar initial weight were concurrently collected via rectal swabbing. The fecal samples of four piglets from one litter were combined into one sample as an experimental unit, such that six process samples (three of each breed) were collected at each time point. All of the fecal samples were collected in the same cryogenic vial and stored in liquid nitrogen.

### DNA Isolation and 16S rRNA Sequencing

Microbial genomic DNA was extracted from 200 mg of each fecal sample using a QIAamp Fast DNA Stool Mini Kit (Qiagen, Valencia, CA, USA) according to the manufacturer's instructions. The following primers were designed to target the V3 and V4 regions of the 16S rRNA based on sequences deposited in the NCBI database: 341F (5′-CCTAYGGGRBGCASCAG-3′) and 806R (5′-GGACTACNNGGGTATCTAAT-3′). The resulting PCR products were extracted from a 2% agarose gel and further purified using a gel DNA extraction kit (Axygen, Union City, CA, USA). Purified PCR products were sequenced on an Illumina MiSeq platform (Illumina, San Diego, CA, USA). Sequencing reads containing more than 10% ambiguous bases (N) or with sequence qualities below 80% (*Q*-value > 20) were filtered from raw data. Filtered reads were subsequently merged using FLASH software with a minimum overlap length of 10 bps and a maximum allowed mismatch ratio of 0.2 in overlapping regions. These merged reads were then subjected to quality control and data optimization using the Trimmomatic software (15). The reads are available in the NCBI SRA database under BioProject accession number PRJNA733567.

### Operational Taxonomic Unit (OTU)-Based Cluster Analysis

Raw sequencing data were subjected to quality control analysis using Quantitative Insights into Microbial Ecology (QIIME software, version 1.17) (16) to trim ambiguous bases or eliminate paired-end reads with combined read lengths greater than the fragment size. Non-duplicate reads were clustered using USEARCH software (version 7.0) (17) into OTUs based on a similarity threshold of 97%. The resulting OTUs were annotated at various taxonomic levels using the Silva (https://www.arb-silva.de/) database (18).

### Analysis of Microbial Diversity

OTUs with 97% similarity were identified in mothur version v.1.30.1 (19). The phylogenetic distance between species and Faith's phylogenetic diversity was determined on the basis of different microbial diversity indices, such as abundance-based coverage estimator (ACE) (http://www.mothur.org/wiki/Ace), Shannon (http://www.mothur.org/wiki/Shannon), and phylogenetic diversity (PD) (https://www.mothur.org/wiki/Phylogenetic_diversity) indices, to estimate alpha (α) diversity of species. Beta (β) diversity was calculated using the R Studio software (v.3.0.2) based on weighted UniFrac distances (20) and principal coordination analysis (PCoA). In addition, OTUs with a similarity of 97% were subjected to rarefaction analysis using the mothur software, and rarefaction curves were plotted using tools in R Studio v.3.0.2 (21).

### Microbial Taxonomic Analysis

A representative sequence for each OTU was analyzed taxonomically using a ribosomal database project (RDP) classifier (RDP naive Bayesian classifier algorithm) (22) to determine the composition of GI microbiota in each sample at phylum, class, order, family, and genus levels. Differences in genus-level abundances of microbes between different age groups and breeds were hypothesis-tested using the Wilcoxon rank-sum test. Breed- and stage-specific genera were subjected to linear discriminant analysis (LDA) using the linear discriminant analysis effect size (LEfSe) software (http://huttenhower.sph.harvard.edu/galaxy/root?tool_id=lefse_upload) to identify microbial genera, which exert significant effects on these two breeds of piglets at various growth stages.

### Statistical Analysis

The resulting body weights and daily weight gains of piglets were presented as mean ± SEM. Statistical analyses were performed using SPSS Statistics 26.0. Differences between Jinfen White pigs and Mashen pigs were analyzed using Student's *t*-test, and differences among different time points per breed were identified using ANOVA. The level of significance was set at *P* < 0.05. Correlations between differential members of the GI microbiota with body weight and average daily weight gain were analyzed using the Vegan R package (http://CRAN.R-project.org/package=vegan), and Spearman's correlation coefficients between environmental factors and selected bacterial genera were calculated.

## Results

### Differences in Growth Rates Between Jinfen White and Mashen Piglets

**Figure 1:**
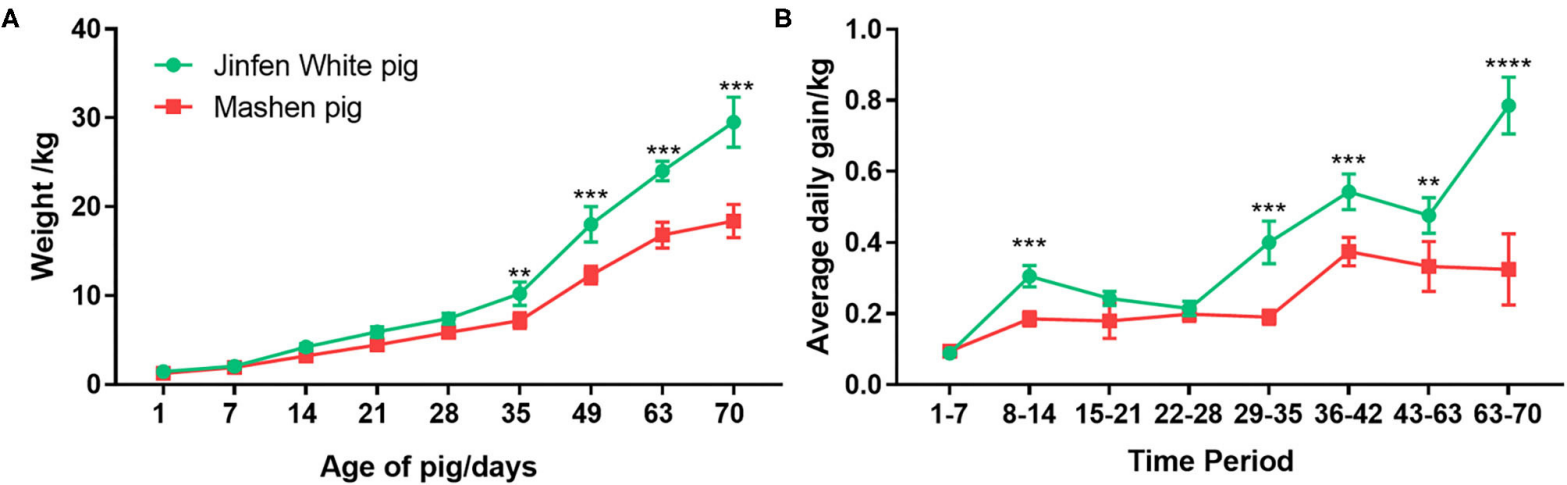
Changes in the growth and development of Jinfen White and Mashen piglets. **(A)** Growth curves for the two breeds of piglets and **(B)** Changes in the average daily weight gain of piglets in each breed. **P < 0.01, ***P < 0.001, and ****P < 0.0001 indicate significant difference from Jinfen White and Mashen piglets.

We weighed piglets every 7 days from days 1 to 70 after farrowing (at the end of the nursery stage) to compare growth rates of Jinfen White and Mashen piglets at different growth stages. As shown in Figure 1A, birth weights of piglets ranged from 1.30 to 1.50 kg, and no significant differences were observed in the body weight between the two breeds from birth (day 1) to weaning (day 28) (*P* < 0.05). Weaned Jinfen White piglets demonstrated significantly higher body weights (*P* < 0.01) than weaned Mashen piglets, especially after 60 days (*P* < 0.001). Average body weights of Jinfen White and Mashen piglets were 29.50 ± 2.81 and 18.37 ± 1.87 kg, respectively, at the end of the nursery stage. Average daily weight gains at each time point were calculated on the basis of differences in body weights between adjacent points to analyze differences in piglet growth rates between the two breeds (Figure 1B). Jinfen White piglets showed a higher average daily weight gain than Mashen piglets from birth through weaning. However, the growth rate of Jinfen White piglets decreased significantly at the end of the suckling stage; otherwise, no significant difference exists in daily weight gain between the two breeds during this period. These two breeds displayed different trends in daily weight gain after entering the nursery stage, with the average daily weight gain of Mashen piglets increasing significantly after 36 days of age although the average daily weight gain of Jinfen White piglets remained significantly higher than that of Mashen piglets at each time point.

### Electrophoresis of PCR Products and Quality Control of 16S rRNA Sequencing Data

**Figure 2:**
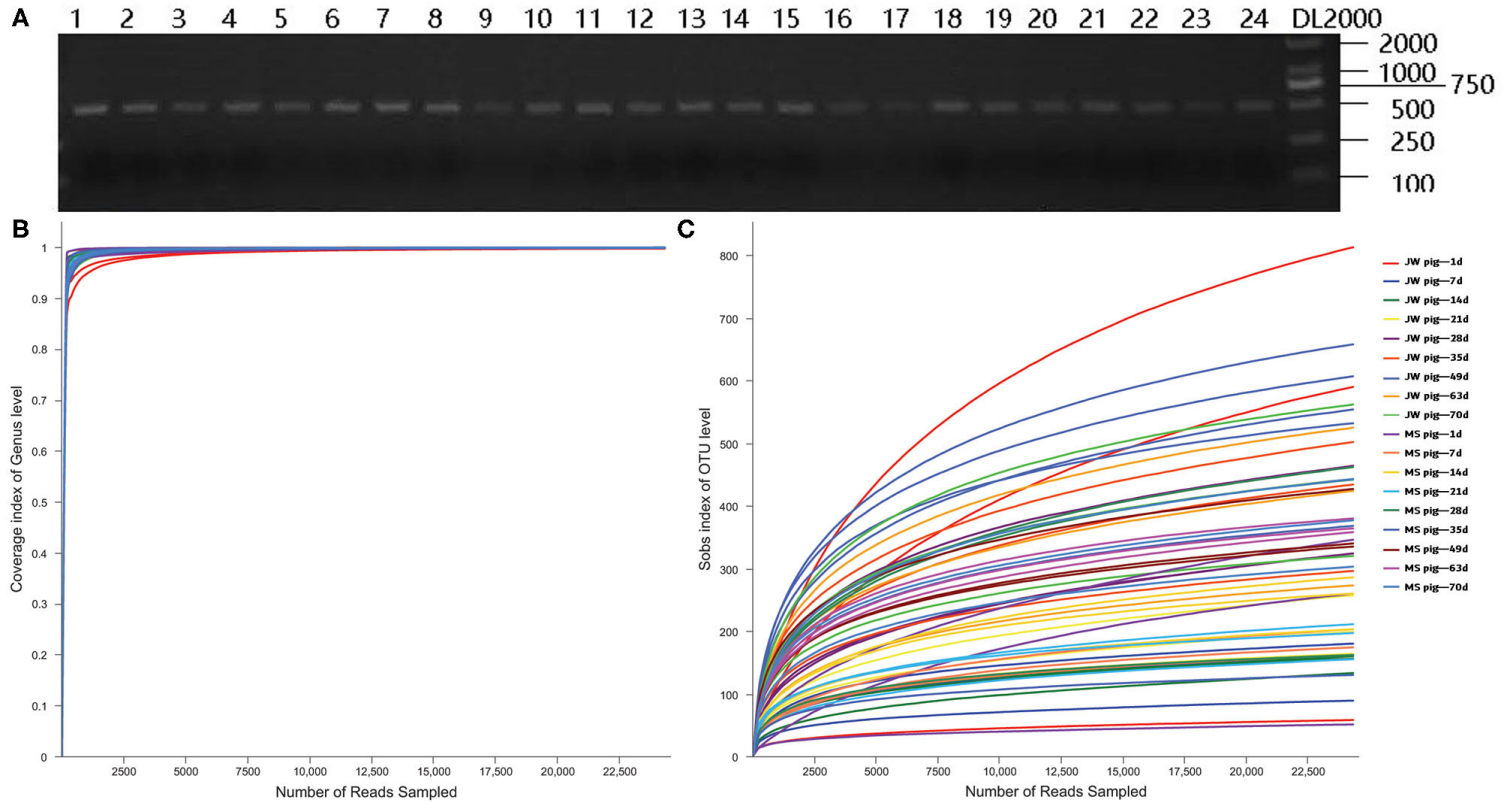
PCR assay with primers targeting 16S rRNA and quality control of the sequencing data. **(A)** Electrophoresis gel image of PCR products amplified using 16S rRNA primers, **(B)** Coverage plots, and **(C)** Sobs rarefaction curves. JB, Jinfen White pigs; MS, Mashen pigs; and F, feces.

Fecal DNA from piglets was used as a template for PCR amplification with 341F and 806R primers, which target 16S rRNA sequences of microorganisms. The PCR assay yielded target bands with correct size and appropriate concentrations for subsequent experiments (Figure 2A). Coverage plots (Figure 2B) and Sobs rarefaction curves (Figure 2C) for each sample constructed in mothur showed a tendency to plateau, thereby indicating that the amount of sequencing data is sufficiently representative of the microbiota diversity in samples.

### OTU-Based Cluster Analysis

Raw sequencing data were normalized to the minimum number of reads to ensure sample homogeneity. OTUs obtained from samples belonged to 27 phyla, 46 classes, 912 orders, 167 families, and 489 genera. A total of 2,881,136 16S rRNA reads (equivalent to 1,258,458,854 bases) with an average length of 437 bps remained after quality filtering (Supplementary Table 1).

### Fecal Microbiota Diversity

**Figure 3:**
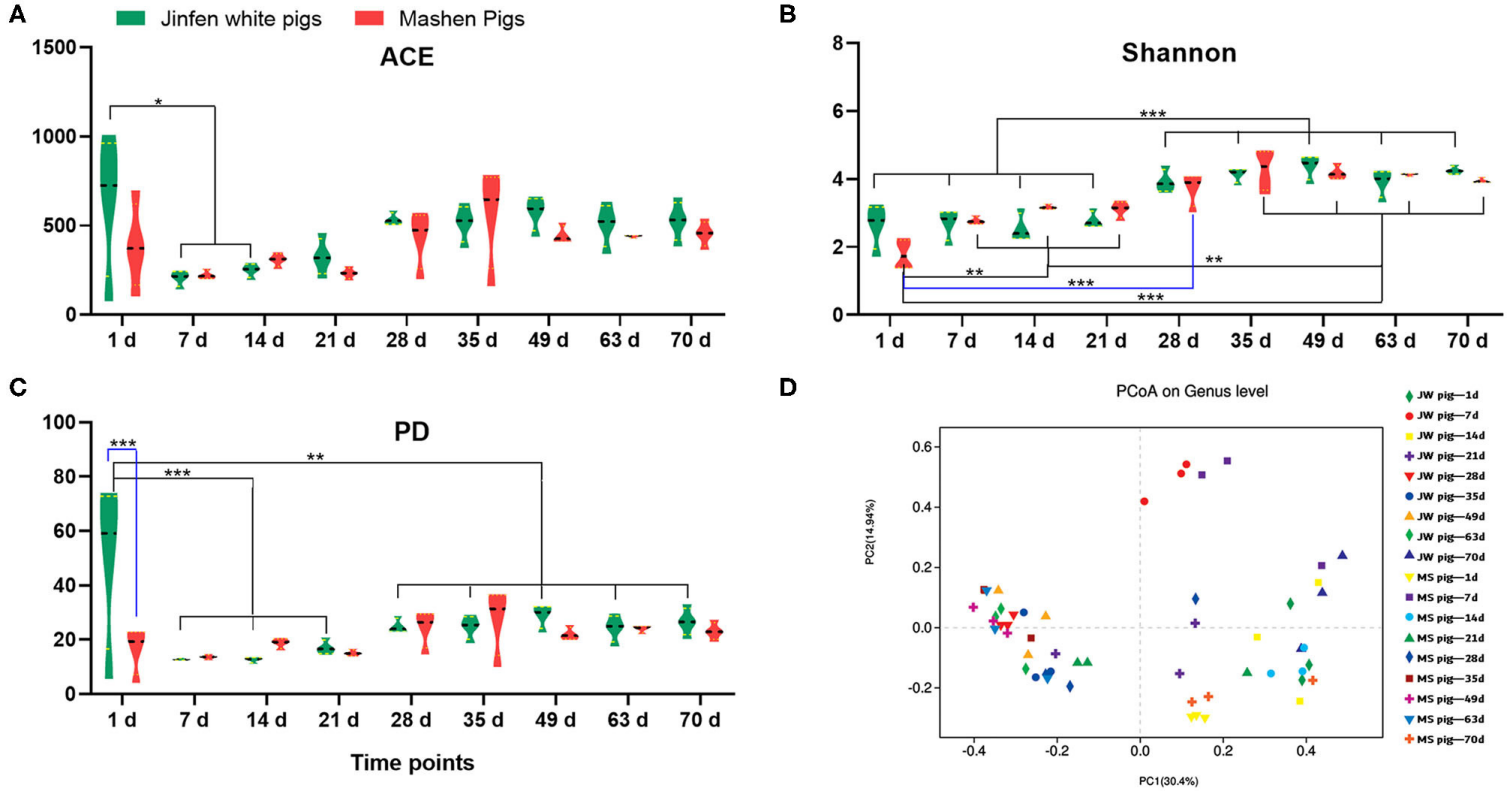
Analysis of the fecal microbiota diversity of Jinfen White and Mashen piglets. **(A)** ACE indices, **(B)** Shannon indices, **(C)** PD indices, and **(D)** PCoA. *P < 0.05, **P < 0.01, and ***P < 0.001 indicate significant difference among different time points.

The α diversity of fecal microbiota from Jinfen White and Mashen piglets was determined on the basis of ACE, Shannon, and PD biodiversity indices. The richness of the fecal microbiota from both breeds of piglets first decreased and then increased with growth and development (Figure 3A). One-day-old Jinfen White piglets showed high fecal microbiota richness that subsequently declined during the suckling stage (*P* < 0.05) before slightly increasing (non-significantly) after piglets entered the nursery stage (*P* > 0.05). Insignificant differences in abundances of microbial components of the fecal microbiota were observed between Mashen and Jinfen White piglets (*P* > 0.05). The Shannon index showed that a significant change exists in fecal microbiota evenness for both piglet breeds (Figure 3B). The evenness of fecal microbiota from Jinfen White piglets was low with insignificant changes from birth (1 day old) through weaning (7, 14, and 21 days old) (*P* > 0.05), followed by a significant increase after weaning (*P* < 0.001); however, the evenness of the fecal microbiota changed non-significantly throughout the nursery stage (*P* > 0.05). Similar to the trend observed for microbiota richness changes, microbial component diversity for the fecal microbiota from the two breeds of piglets first declined and then increased with growth and development (Figure 3C). Newborn Jinfen White piglets demonstrated the maximum fecal microbiota diversity, and this diversity was significantly higher than that at the suckling, weaning, and nursery stages (*P* < 0.001). Microbiota diversity first decreased significantly at the suckling stage and then increased after weaning (*P* < 0.05), before stabilizing at the nursery stage. Similar to fecal microbiota diversity in Jinfen White piglets, that of Mashen piglets also first decreased and then increased, but no significant changes were observed across all growth stages (*P* > 0.05). Jinfen White piglets showed greater fecal microbiota diversity than Mashen piglets at birth (*P* < 0.001).

PCoA (Figure 3D) showed that samples from the two breeds of piglets can be aggregated into three clusters. The first cluster in the top-left quadrant comprises samples from 1-day-old piglets, which show a greater degree of dispersion than other age groups. The second cluster in the top-right quadrant of the PCoA chart comprises samples from 7- to 21-day-old piglets clustered together but with a relatively low degree of aggregation. The third cluster in the bottom-right quadrant comprises samples from 28-day-old weaned piglets with GI microbiota that showed a high degree of change in response to unexpected dietary shifts, whereby piglets were given solid feed pellets with poor taste in place of breast milk, which is easily digested and absorbed. The last cluster in the left-bottom quadrant of the PCoA chart comprises samples from 35- to 70-day-old piglets. These samples were clustered together with a relatively high degree of aggregation. Overall, these results demonstrated that the β diversity of the piglet fecal microbiota increased with age and was primarily unrelated to breed.

### GI Microbiota Composition in Piglets at Various Growth Stages

**Figure 4:**
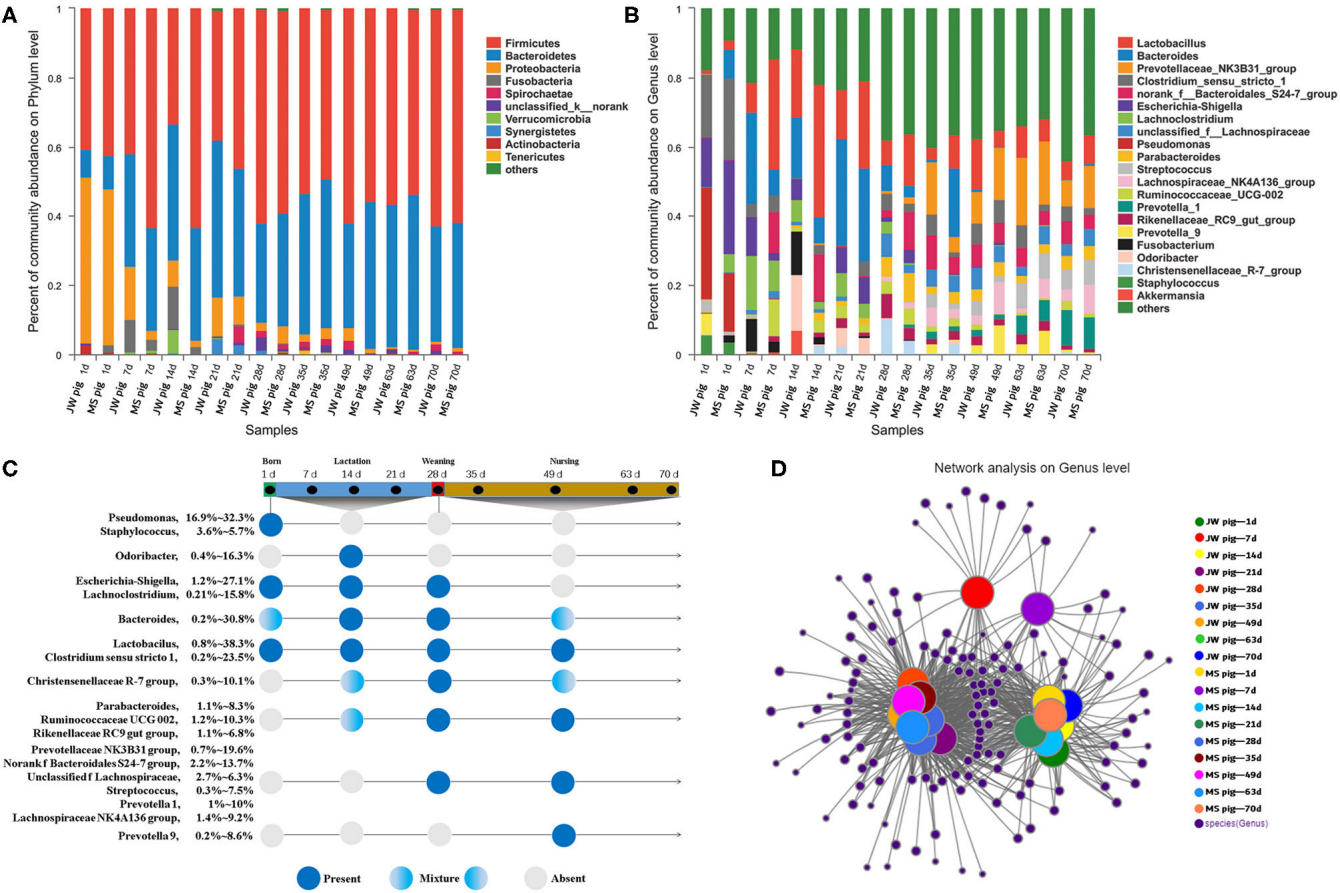
Characterization of fecal microbiota from piglets. **(A)** Microbial community bar plot of phyla in piglet feces. **(B)** Microbial community bar plot of genera in piglet feces. **(C)** Longitudinal patterns in piglet fecal microbiomes based on averaged relative abundances according to days after birth. **(D)** Network analysis of microbial co-occurrences in piglet feces.

We analyzed the fecal microbiota composition and the time of first appearance of OTUs in piglets at various developmental stages (Supplementary Table 2). Our study identified 27 phyla in the fecal microbiota. Among them, Firmicutes was the most abundant (33.2–63.2%), followed by Bacteroidetes (8.2–45.1%). The abundance of Bacteroidetes in feces gradually increased with piglet age. By comparison, Proteobacteria was the most abundant (45.1–47.8%) at birth and then decreased significantly to <1% of the total fetal microbiota at the late nursery stage (Figure 4A).

OTUs (1,976) discovered in this study were annotated into 489 genera. Among these, *Lactobacillus, Bacteroides*, and *Prevotellaceae* NK3B31 groups were the most abundant at 12.4, 8.8, and 5.8% of the total fetal microbiota, respectively (Figure 4B). The 20 most abundant genera were classified by order and time of first appearance (Figure 4C). *Pseudomonas* and *Staphylococcus* were the most abundant only at birth, while *Odoribacter* was the most abundant in suckling piglets but significantly less abundant at subsequent stages. *Escherichia–Shigella* and *Lachnoclostridium* were detected in suckling piglets but disappeared gradually after weaning. *Lactobacillus* and *Clostridium sensu stricto 1* were detected in piglets across growth stages. *Parabacteroides* and other genera gradually increased in abundance in piglets after 7 days of age and were detected in suckling, weaning, and nursery piglets. *Streptococcus* and other genera predominated in the fecal tracts of weaned piglets, while *Prevotella* 9 was consistently detected in piglets during the late nursery stage.

### Co-occurrence Network Analysis of Piglet Microbiota at Various Developmental Stages

Only OTUs >50 were subjected to co-occurrence network analysis to reduce network complexity. The results are shown in Figure 4D. The co-occurrence network shown includes 371 genera shared among 54 samples, which were collected at nine time points from piglets of each breed. Each shared genus was scored for its weighted degree. The top 20 abundant genera, except for *Staphylococcus*, scored above 5,000 with a node tightness >0.5 (Supplementary Table 3). The co-occurrence network contained 51 core genera, including *Lactobacillus, Bacteroides*, and *Prevotella 2*, with 30 genera, including *Escherichia-Shigella, Globicatella, Faecalibacterium*, and *Pseudomonas*, shared among 1-day-old piglets. Four unique genera (*Negativicoccus, Holdemania*, unclassified f *Veillonellaceae*, and *Lachnospiraceae* UCG-006) were detected in feces from 7- to 21-day-old piglets. In addition, 21 genera (e.g., *Lachnospiraceae* NK4B4 group, *Prevotella 1*, and *Ruminococcaceae* V9D2013 group) were detected in feces from 28- to 70-day-old piglets (Supplementary Table 4). Fecal samples collected from 1- to 70-day-old piglets were divided into the following stages on the basis of the co-occurrence of bacteria in different samples: stage 1, composed of fecal samples collected from 1-day-old Jinfen White and Mashen piglets; stage 2, composed of fecal samples collected from 7- to 21-day-old piglets; and stage 3, composed of fecal samples collected from 28- to 70-day-old piglets. These results were consistent with those determined via PCoA.

### Differential Microbiota Compositions in Jinfen White and Mashen Piglets Across Growth Stages

**Figure 5:**
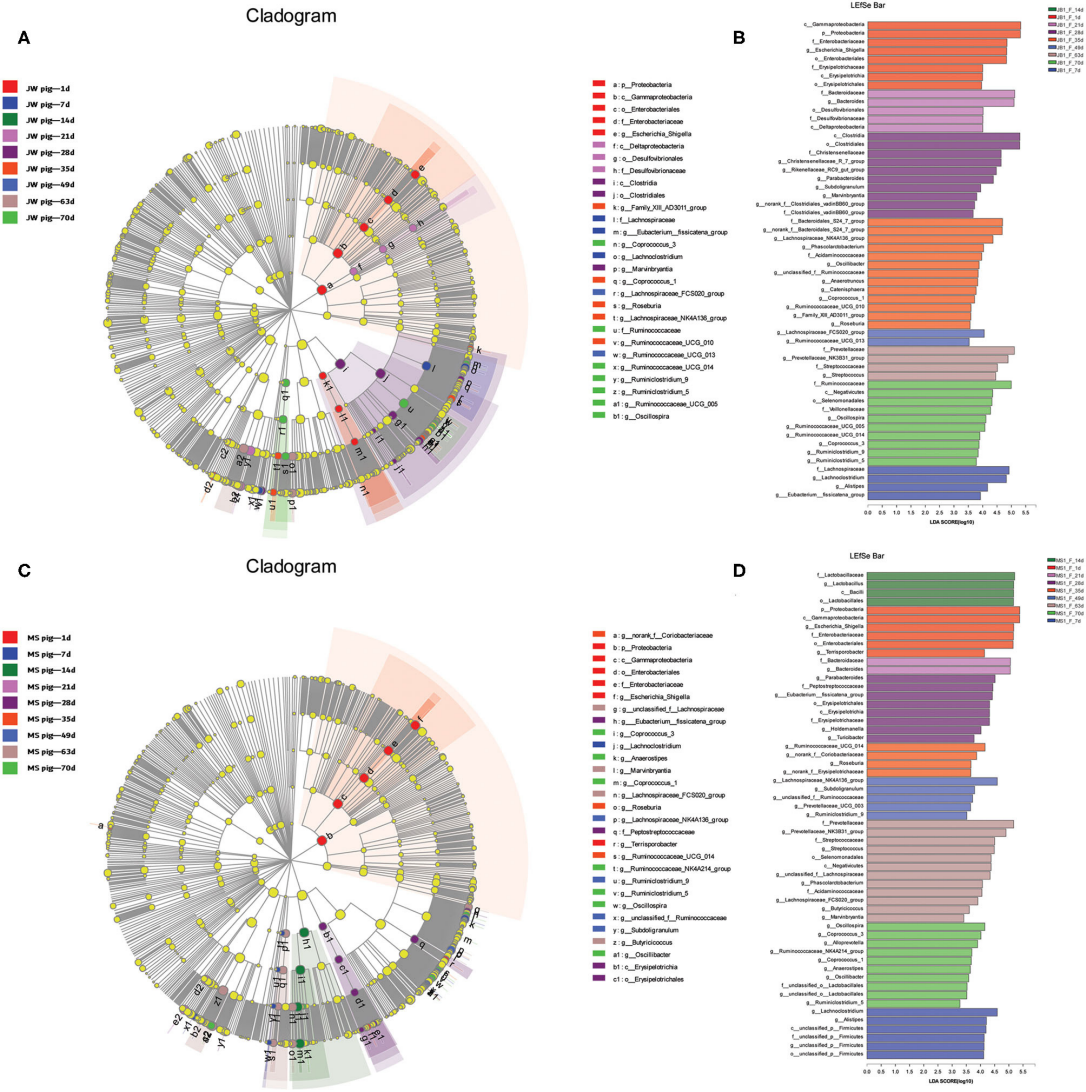
Beta diversity of bacterial communities in feces of piglets at different development stages. **(A,B)** Linear discriminatory analyses (LEfSe) of bacterial taxa in Jinfen White piglets (LDA > 3). **(C,D)** Linear discriminatory analyses (LEfSe) of bacterial taxa in Mashen piglets (LDA > 3).

The fecal microbiota from the two breeds of piglets at different growth stages were analyzed via LEfSe multilevel species discrimination. Fifty-four genera were identified (Figure 5 and Supplementary Tables 5, 6). The genera *Escherichia* and *Shigella* were shared between newborn Jinfen White and Mashen piglets. In addition, *Erysipelotrichaceae* and *Terrisporobacter* were specifically found in Jinfen White and Mashen piglets, respectively. *Bacteroides, Desulfovibrionaceae, Lachnoclostridium, Alistipes*, and the *Eubacterium fissicatena* group were specifically found in suckling Jinfen White piglets, whereas only three genera of *Lactobacillus, Bacteroides*, and unclassified p *Firmicutes* were specifically found in suckling Mashen piglets. *Bacteroides* was the only genus shared between the two breeds of piglets at the suckling stage. Seventeen genera (*Marvinbryantia, Subdoligranulum, Parabacteroides, Roseburia, Coprococcus 1*, unclassified f *Ruminococcaceae, Oscillibacter, Phascolarctobacterium, Lachnospiraceae* NK4A136 group, *Lachnospiraceae* FCS020 group, *Streptococcus, Prevotellaceae* NK3B31 group, *Ruminiclostridium* 5, *Ruminiclostridium* 9, *Coprococcus* 3, *Ruminococcaceae* UCG 14, and *Oscillospira*) were shared between the two breeds of piglets from weaning to the end of the nursery stage. In addition to these 17 genera, 12 (no rank f *Clostridiales vadin* BB60 group, *Rikenellaceae* RC9 gut group, *Christensenellaceae* R 7 group, Family XII AD3011 group, *Ruminococcaceae* UCG 10, *Catenisphaera, Anaerotruncus, Acidaminococcaceae*, no rank f *Bacteroidales* S24 7 group, *Ruminococcaceae* UCG 13, *Ruminococcaceae* UCG 5, and *Veillonellaceae*) and 14 (*Peptostreptococcaceae, Eubacterium fissicatena* group, *Holdemanella, Turicibacter*, no rank f *Coriobacteriaceae*, no rank f *Erysipelotrichaceae, Prevotellaceae* UCG 3, *Selenomonadales*, unclassified f *Lachnospiraceae, Butyricicoccus, Alloprevotella, Ruminococcaceae* NK4A214 group, *Anaerostipes*, and unclassified o *Lactobacillales*) genera were specifically found in Jinfen White and Mashen piglets from weaning to the end of the nursery stage.

### Correlations Between Microbial Diversity and Piglet Growth Rate

**Figure 6:**
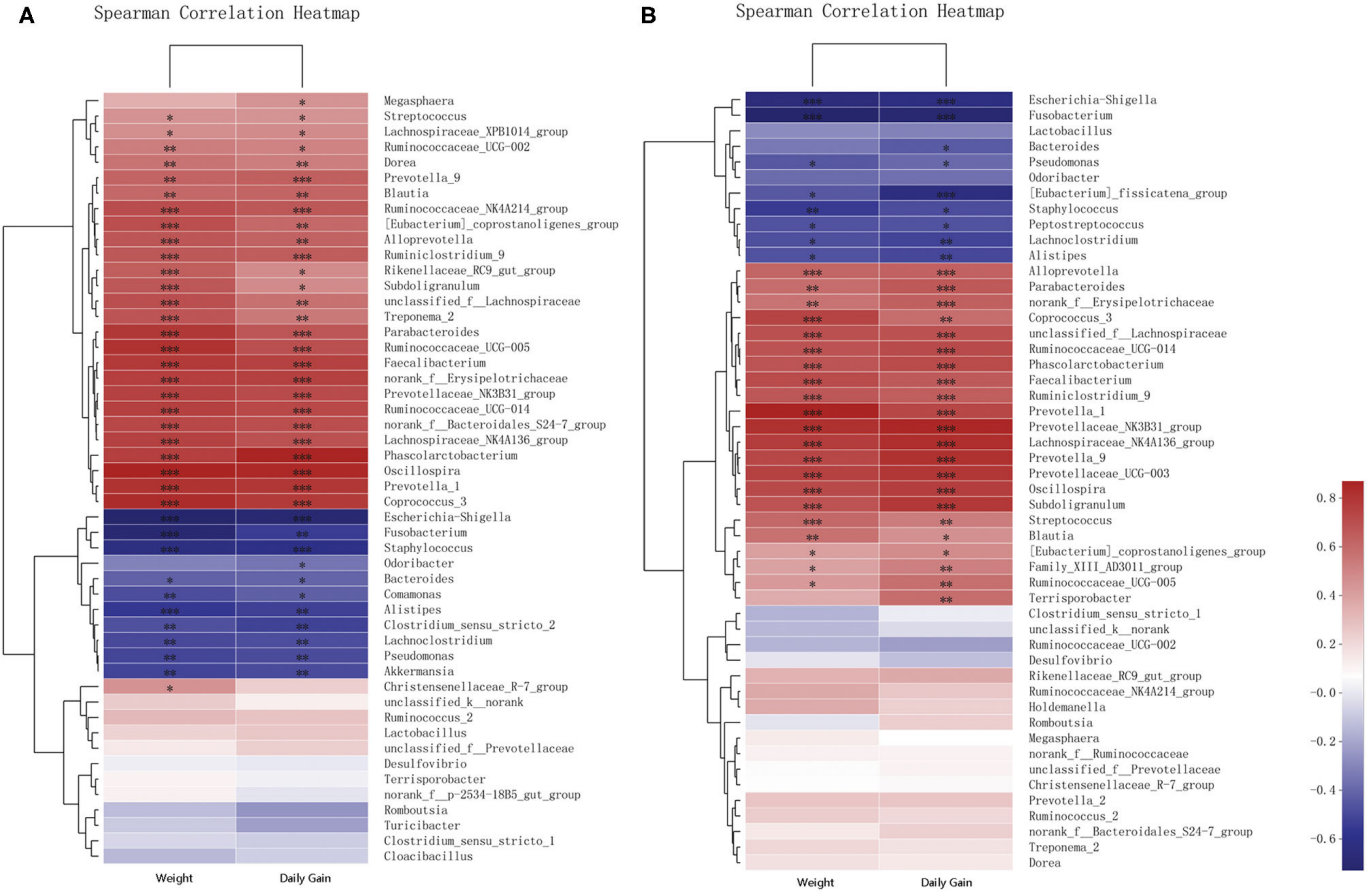
Correlation analysis of genera and environmental variables. **(A)** Correlation between Jinfen White piglets and environmental variables and **(B)** Correlation between Mashen piglets and environmental variables. The relationships between microbial genera found in samples and environmental variables are displayed in heatmaps with correlation coefficients. Columns and rows represent environmental factors and genera, respectively. *R*- and *P*-values were calculated. *R*-values are represented by colors, as indicated in the figure legend on the right side of the figure. JBW, Body weight of Jinfen White piglets and MSW, Body weight of Mashen piglets. *P < 0.05, **P < 0.01, and ***P < 0.001 indicate significant correlation between microbial genera and weight or daily gain.

Correlations of the top 50 abundant genera in feces from the two breeds of piglets with piglet body weights and daily weight gains were determined by calculating Spearman's correlation coefficients (Figure 6 and Supplementary Tables 7, 8). The correlation analysis revealed 41 genera, including *Oscillospira, Phascolarctobacterium*, and *Escherichia–Shigella*, which showed significant correlations with body weights. Body weights of piglets were significantly positively correlated with abundances of 29 genera, including *Oscillospira, Phascolarctobacterium*, and *Prevotellaceae* NK3B31 groups. Among them, 19 genera were shared between the two breeds of piglets, with eight and two genera specific to Jinfen White (Figure 6A) and Mashen (Figure 6B) piglets, respectively. However, body weights of piglets were significantly negatively correlated with abundances of 12 genera, including *Escherichia–Shigella, Fusobacterium*, and *Staphylococcus*. Amongst them, six genera (i.e., *Lachnoclostridium, Pseudomonas, Alistipes, Fusobacterium, Staphylococcus*, and *Escherichia–Shigella*) were shared between the two breeds of piglets, with four (i.e., *Bacteroides, Comamonas, Akkermansia*, and *Clostridium sensu stricto* 2) and two genera specific to Jinfen White (Figure 6A) and Mashen (Figure 6B) piglets, respectively.

The analysis showed that 43 genera were significantly correlated with daily weight gains. Nearly all genera that showed significant correlations with body weights also presented significant correlations with daily weight gains. Twenty-five genera displayed significant correlations with both body weights and daily weight gains of piglets. The abundance of *Bacteroides* correlated significantly with daily weight gains of piglets of both breeds and body weights of Jinfen White piglets; however, no significant correlation was observed with body weights of Mashen piglets. Feces of Jinfen White piglets contained 10 genera significantly correlated with body weights and daily weight gains, but no significant correlation existed between these genera and body weights and daily weight gains of Mashen piglets. Similarly, feces of Mashen piglets contained four genera significantly correlated with body weights and daily weight gain, but no significant correlation was observed between these genera and body weights and daily weight gains of Jinfen White piglets.

## Discussion

Piglets progress through critical stages of growth and development and showed distinct physiological, nutritional, metabolic, and thermoregulatory profiles during these stages. The GI microbiota flora and composition of piglets hinder piglet growth and development due to their sensitivity and vulnerability to environmental conditions. Different microbiota profiles require unique nutritional requirements and breeding plans and can signal nutritional disorders (e.g., diarrhea) in piglets. The digestive organs of piglets are formed but not fully developed structurally and functionally during the embryonic stage. Piglets exhibit poor non-dairy carbohydrate utilization despite high intestinal lactase activity. The GI tract of pigs is gradually colonized by microorganisms as they grow and develop. Therefore, a study that examines changes in the GI microbiota composition is important for the management of piglet feeding.

The GI microbiota in animals are subject to temporal and spatial changes. These microorganisms that compose the microbiota play important roles in establishing a stable microecosystem. A balanced and stable microbiota structure is a prerequisite for animal health, but microbiota structure may be affected by numerous factors, including the animal's breed, dietary pattern, age, and health condition (23, 24). We examined the GI microbiota of two local pig breeds, Mashen and Jinfen White (crossbred from Mashen pigs), over the course of 70 days in this study. Overall, our results demonstrated that only a small difference existed in fecal microbiota composition while significant differences in growth rate occurred between the piglet breeds. Microbiota richness and diversity first decreased and then increased along with growth and development of piglets and remained mostly stable during the nursery stage (28–70 days old). Our results are consistent with those of Niu et al. (25), wherein richness and diversity of the GI microbiota in pigs are positively correlated with age. We performed PCoA on the basis of weighted UniFrac distances to understand how the GI microbiota in Jinfen White and Mashen piglets change with growth further. Piglets were divided into the following stages based on fecal microbiota diversity: immediate postfarrowing (1 day old), suckling (7–21 days old), and nursery (28–70 days old) stages. Similar patterns were observed in the OTU-based co-occurrence network analysis. Numerous studies have suggested that Firmicutes and Bacteroidetes are the core phyla in GI tracts of mammals (26, 27). Zhao et al. (28) revealed that Firmicutes is the dominant phylum in feces, with a trend toward increasing abundance with age (in days) of piglets, whereas Proteobacteria shows the opposite trend, with greater abundance in younger (in months) piglets. Our results demonstrated that the abundances of Firmicutes and Bacteroidetes in feces of Jinfen White and Mashen piglets remain high across multiple growth stages. In addition, Bacteroidetes abundance increased gradually with piglet growth while Proteobacteria demonstrated maximum abundance at birth. Our results are consistent with the findings of Zhao et al. which indicated the maximum abundance of Proteobacteria at birth before it begins to decrease after 1 month of age.

Here, we analyzed the order of first appearance of microorganisms in the GI microbiota of piglets to determine the role for these bacteria in growth and development. Abundances of *Clostridium sensu stricto* 1, S*taphylococcus, Escherichia–Shigella, Globicatella, Faecalibacterium*, and *Pseudomonas* in feces from newborn piglets were elevated but decreased significantly or even disappeared at 7 days of age. Diarrhea in newborn piglets may be attributed to the presence of certain bacteria in the microbiota, such as *Escherichia–Shigella* and *Pseudomonas* (29, 30), which are often transferred from the body, vagina, or amniotic fluid of the mother sow or delivery beds and the air. Accordingly, Proteobacteria are abundant in feces of piglets at birth. The origin of these organisms should be investigated in future studies because our work mainly focused on changes in the composition of the GI microbiota in piglets after farrowing. We also found that *Odoribacter* is abundant in suckling piglet feces. These bacteria are important Bacteroidetes members that can produce butyrate, which is associated with lipid metabolism in animals and contributes to intestinal motor function by targeting the enteric nervous system (31). The gradual increase of abundance of the *Lachnospiraceae* NK4A136 group in piglets after weaning is closely associated with energy production and butyrate metabolism (32). The *Prevotellaceae* NK3B31 group, which predominated in GI tracts of nursery piglets, is involved in starch degradation and glucose metabolism, helps provide the body with energy for growth and development, and maintains a balanced and healthy GI tract (33). The abundance of *Prevotella* 9 gradually increased and then remained stable in nursery piglets. Previous studies revealed that supplementation of piglet feed with *Bacillus licheniformis*-fermented products significantly reduces the rate of diarrhea among weaned piglets and that the average abundance of *Prevotella* 9 in feces correlates positively with *B. licheniformis*-fermented products, thereby suggesting that these bacteria can improve piglet productivity (34). Our study also showed that *Lactobacillus* is present in all fecal samples from both breeds of piglets. *Lactobacillus* promotes piglet early development and can improve the GI health of newborn piglets by regulating GI microbiota (35–37).

Body weights of piglets correlated positively with the abundance of genera, such as *Subdoligranulum, Prevotellaceae* NK3B31 group, *Prevotella* 9, and *Bacteroidales* S24-7 group, in this study. *Subdoligranulum* produces short-chain fatty acids, which can reduce inflammation by decreasing intestinal permeability, and thus promotes piglet growth and development (38). The *Prevotellaceae* NK3B31 group and *Prevotella* 9, which belong to the genus *Prevotella*, also exhibit a growth-promoting effect because they can reduce inflammation by decreasing intestinal permeability (38). In addition, both the *Prevotellaceae* NK3B31 group and *Prevotella* 9 can aid in degrading proteins and carbohydrates found in foods, producing intramuscular fats and storing hepatic glycogen, which promotes body weight gain in pigs (11, 39). The *Bacteroidales* S24-7 group also plays an important role in maintaining GI health by inhibiting GI inflammation (40). *Akkermansia, Escherichia–Shigella*, and *Fusobacteria* showed strong negative correlations with body weight gain in the two breeds of piglets. *Akkermansia*, which is highly abundant at the early growth stages, is a member of the Verrucomicrobia phylum, and its presence is negatively correlated with the body weight gain of animals (41). *Fusobacteria* are more abundant in suckling piglets than in piglets in other growth stages. *Fusobacteria* and other pathogenic bacteria often found in GI tracts of suckling piglets may cause diseases such as diarrhea and enteritis (42).

Overall, our results suggested that the composition of the GI microbiota can significantly affect the growth and development of piglets. Environmental control of bacterial exposures for newborn piglets should be considered in pig production. Creep feeding before weaning reduces stress caused by weaning and rapid replacement feeding and can help maintain GI microbiota stability in piglets to a certain extent, thereby reducing onset of diarrhea and improving piglet growth.

## Conclusions

Microbiota compositions of Jinfen White and Mashen piglets varied significantly across stages. Fecal samples from piglets were divided into immediate postfarrowing, suckling, and nursery stages on the basis of fecal microbiota diversity. The GI microbiota in newborn piglets is significantly affected by maternal and environmental factors. High abundances of pathogenic bacteria, such as *Akkermansia, Escherichia–Shigella*, and *Fusobacteria*, may cause diseases (e.g., diarrhea) in piglets. An abundance of beneficial bacteria, such as *Lactobacillus, Subdoligranulum, Prevotellaceae* NK3B31 group, *Prevotella 9*, and *Bacteroidales* S24-7 group, displayed a strong positive correlation with body weight and daily weight gain in piglets. Our results demonstrate succession patterns in the fecal microbiota of piglets and can provide a theoretical basis for piglet feeding management to improve growth rates for pig production.

## Data Availability Statement

The datasets presented in this study can be found in online repositories. The names of the repository/repositories and accession number(s) can be found below: Sequence Read Archive (PRJNA733567).

## Ethics Statement

The animal study was reviewed and approved by the Animal Ethics Committee of Shanxi Agricultural University.

## Author Contributions

YY and YL analyzed the data and drafted the manuscript under the supervision of PG and MD. JL and YG carried out the bioinformatics analyses, under the supervision of GC and BL. ZD and PG conceived the study. HW prepared the DNA samples. CC performed the PCR studies. YZ and CL carried out 16S rRNA-seq mapping, under the supervision of XG. All authors have read and approved the final manuscript.

## Conflict of Interest

The authors declare that the research was conducted in the absence of any commercial or financial relationships that could be construed as a potential conflict of interest.
